# Copy Number Variants in Extended Autism Spectrum Disorder Families Reveal Candidates Potentially Involved in Autism Risk

**DOI:** 10.1371/journal.pone.0026049

**Published:** 2011-10-07

**Authors:** Daria Salyakina, Holly N. Cukier, Joycelyn M. Lee, Stephanie Sacharow, Laura D. Nations, Deqiong Ma, James M. Jaworski, Ioanna Konidari, Patrice L. Whitehead, Harry H. Wright, Ruth K. Abramson, Scott M. Williams, Ramkumar Menon, Jonathan L. Haines, John R. Gilbert, Michael L. Cuccaro, Margaret A. Pericak-Vance

**Affiliations:** 1 John P. Hussman Institute for Human Genomics, Miller School of Medicine, University of Miami, Miami, Florida, United States of America; 2 Dr. John T. Macdonald Foundation Department of Human Genetics, Miller School of Medicine, University of Miami, Miami, Florida, United States of America; 3 Department of Neuropsychiatry, University of South Carolina School of Medicine, Columbia, South Carolina, United States of America; 4 Center for Human Genetics Research, Vanderbilt University, Nashville, Tennessee, United States of America; 5 Department of Epidemiology and Department of Obstetrics and Gynecology, Rollins School of Public Health, Emory University, Atlanta, Georgia, United States of America; Emory University School Of Medicine, United States of America

## Abstract

Copy number variations (CNVs) are a major cause of genetic disruption in the human genome with far more nucleotides being altered by duplications and deletions than by single nucleotide polymorphisms (SNPs). In the multifaceted etiology of autism spectrum disorders (ASDs), CNVs appear to contribute significantly to our understanding of the pathogenesis of this complex disease. A unique resource of 42 extended ASD families was genotyped for over 1 million SNPs to detect CNVs that may contribute to ASD susceptibility. Each family has at least one avuncular or cousin pair with ASD. Families were then evaluated for co-segregation of CNVs in ASD patients. We identified a total of five deletions and seven duplications in eleven families that co-segregated with ASD. Two of the CNVs overlap with regions on 7p21.3 and 15q24.1 that have been previously reported in ASD individuals and two additional CNVs on 3p26.3 and 12q24.32 occur near regions associated with schizophrenia. These findings provide further evidence for the involvement of *ICA1* and *NXPH1* on 7p21.3 in ASD susceptibility and highlight novel ASD candidates, including *CHL1, FGFBP3* and *POUF41*. These studies highlight the power of using extended families for gene discovery in traits with a complex etiology.

## Introduction

Autism spectrum disorders (ASDs) encompass a range of abnormal neurodevelopmental phenotypes characterized by a core set of symptoms including social, communicative and behavioral impairments [1]. The most recent prevalence studies suggest that ASDs affect as many as 1 in 110 children [2]–[4]. While ASD etiology is multifaceted, a significant portion is known to stem from genetic causes given the increased concordance rate in monozygotic twins (∼91%) as compared to dizygotic twins (∼10%) [5]–[8]. Genetic studies over the past decade have demonstrated that the underlying components are complex with multiple genes acting either independently or perhaps synergistically to result in ASD phenotypes. While hundreds of candidate genes have been published, linkage and association studies have only recognized a small number of consensus regions [9]. Additionally, genome-wide association studies thus far have identified very few regions that may contain a common genetic cause, such as the regions on 5p14.1 and 5p15 [10]–[12]. Therefore, it appears that rare and private variations may represent a considerable portion of the genetic components involved in ASDs.

A growing number of studies have demonstrated the potential contribution of copy number variations (CNVs) in the etiology of many complex disorders including autism, Alzheimer's disease, epilepsy, and schizophrenia[13], [14]. To date, CNVs believed to be involved in ASDs occur at a relatively low frequency and are often private, familial alterations, reflecting the genetic heterogeneity of this disorder [15]. Furthermore, these alterations have implicated a variety of genes within the glutamatergic, ubiquitin, and cell adhesion pathways [16], [17]. However, only a limited number of findings have been replicated between research groups including CNVs in *CNTN4*, *NRXN1*, 15q11-q13, 16p11.2, and 22q11.21 [17]–[20]. In addition, there appears to be variability in the clinical consequences of CNVs. For instance, patients with CNVs on 16p11.2 have been identified with a range of features including autism, developmental delay, intellectual disabilities and schizophrenia [19], [21]–[23]. Furthermore, there is evidence of phenotypic heterogeneity within families carrying a 16p11.2 CNV including siblings discordant for autism and transmission of a CNV from asymptomatic parents [24], [25]. This demonstrates that while a CNV may provide a strong susceptibility to a phenotype, it may act in concert with other genetic and environmental factors.

Several studies suggest that familial and sporadic cases of ASD are genetically distinct. Extended, multiplex families are more likely to carry heritable risk factors while, in contrast, sporadic ASD families have demonstrated a higher rate of *de novo* CNVs [26], [27]. In this study, we examine 42 unique extended ASD families and identify rare structural alterations that are shared between multiple affected individuals. We hypothesized that that we would unearth novel ASD susceptibility genes with a moderate to strong effect by identifying CNVs that segregate in distantly affected relatives. Intriguingly, many CNVs carriers, while not being clinically diagnosed with an ASD, nonetheless present with a history of psychiatric and developmental conditions. Our study demonstrates the importance of decoding the various genetic components involved in ASDs.

## Materials and Methods

### Ethics statement

We ascertained individuals at the John P. Hussman Institute for Human Genomics (HIHG) at the University of Miami Miller School of Medicine (Miami, Florida), the University of South Carolina (Columbia, South Carolina), and the Center for Human Genetics Research at Vanderbilt University (Nashville, Tennessee). Written informed consent was obtained from parents for all minor children and those who were unable to give consent. In addition, we obtained assent from all participants of the appropriate developmental and chronological age. All participants were ascertained using the protocol approved by the appropriate Institutional Review Boards. Patients were collected for this study for over a decade, with protocols and amendments being approved at each stage. Oversight of the study at the University of Miami falls under the UM Medical IRB B committee whose members includes Ofelia Alvarez, M.D., Abdul Mian, Ph.D., Maria Alcaide, M.D., Michelle Dunaj Lucking, J.D., Jean Jose, D.O., Howard Landy, M.D., Bruce Nolan, M.D., FACP, FAASM, Emilio Weiss, Pharm.D., and Cecilia Grano de Oro, B.A.

### Ascertainment and sample description

Our dataset consisted of 410 individuals from 42 extended ASD families. Extended families were defined as families with at least two affected cousins. These families included either two (n = 28), three (n = 8) or four (n = 6) cousins or more distant relatives with ASDs. We collected DNA from all affected and unaffected family members wishing to participate.

ASD families were enrolled and recruited via support groups, advertisements, and from clinical and educational settings. Core inclusion criteria for ASD individuals were as follows: (1) chronological age between 3 and 21 years of age, (2) a presumptive clinical diagnosis of ASD, (3) an expert clinical determination of ASD diagnosis using DSM-IV criteria supported by the Autism Diagnostic Interview (ADI-R) [28], [29], and (4) an IQ equivalent > 35 or developmental level >18 months as determined by the Vineland Adaptive Behavior Scale (VABS) [30]. Diagnostic determination was based on review by a panel consisting of experienced clinical psychologists and a pediatric medical geneticist. In those instances where an ADI-R was not available (n = 18), a best-estimate diagnosis was assigned using all available clinical information including clinician summaries, a caregiver report, and medical records. We excluded participants with severe sensory problems (e.g., visual impairment or hearing loss), significant motor impairments (e.g., failure to sit by 12 months or walk by 24 months), or identified metabolic, genetic, or progressive neurological disorders. Family history and pedigree information was collected in a standard semi-structured interview from a knowledgeable informant, most frequently the mother. Additional clinical data were also collected by reviewing available medical and psychiatric records of affected individuals.

Ancestry was identified by self-report and subsequently confirmed by genotyping data plotted in EIGENSTRAT and compared with HapMap populations [31], [32]. One family, 7606, was of African American descent and 41 families were of European descent. Thirty-one families had only male ASD individuals while the remaining eleven families had both male and female affected individuals. Genomic DNA used for CNV identification was purified from three different sources, whole blood (n = 276), saliva (n = 8) and cell lines (n = 126), using Puregene chemistry on the Qiagen Autopure LS according to standard protocols. Sample concentration was assessed via the NanoDrop 8000 Spectrophotometer (Thermo Scientific) and all samples were normalized to 50 ng/ul prior to genotyping.

### Control individuals

Eight hundred and ninety-nine healthy children were recruited by two centers. The first cohort was recruited by the Hussman Institute for Human Genomics (HIHG) at the University of Miami and included 565 children between the ages of 4 and 21 years. Participants with developmental, behavioral, or neurological conditions were excluded, as well as those with first degree relatives with such disorders. We obtained consent from all participants or, in the case of minors, their parents. Participants meeting these criteria provided a saliva sample. A knowledgeable informant, usually the mother, completed the Social Communication Questionnaire to screen for potential ASDs [33]. The remaining 334 control individuals were part of a preterm birth study from Nashville (Nashville Birth Cohort, NBC). These samples were collected from the Centennial Medical Center in Nashville, Tennessee from the cord blood of term pregnancies (> 37 weeks gestation) and were not screened for ASDs. All samples were collected from live, singleton births delivered by mothers between the ages of 18 and 40.

Race was identified in both control cohorts by parental self-report and verified by comparing the genotyping data to HapMap populations in EIGENSTRAT [31], [32]. In rare cases of inconsistency, the genetically determined ancestry was used. In total, 112 controls were classified as African American and 787 of European descent.

### Genotyping and copy number variation discovery

Genotyping of extended family members was performed with either the Illumina Human 1Mv1_C BeadChip (n = 349) or the 1M-DuoV3 BeadChip (n = 61). Samples were processed using the Infinium II assay. After genotyping, we carried out a series of quality control (QC) tests. Reported and genetic sex were examined using X-chromosome linked SNPs. Relatedness between samples and potential sample contaminations were evaluated using genome-wide identity by descent and identity by state estimation through genotyped SNPs in PLINK (http://pngu.mgh.harvard.edu/~purcell/plink/ibdibs.shtml). We tested for Mendelian inconsistencies for all SNPs in the WASP/PLATO software (https://chgr.mc.vanderbilt.edu/plato). Samples with gender inconsistencies, elevated Mendelian error rates and genotyping call rates below 95% were removed. All controls were genotyped on the Human 1 M-DuoV3 BeadChips and underwent the same QC steps as the family cohort with exception of testing for Mendelian errors.

All subjects that passed QC were entered into the CNV analysis. The CNV calls were generated via the PennCNV algorithm (August 2009 version) using Log R ratio (LRR) and B allele frequency [34]. Individual CNV calling was adjusted for intensity wave artifacts (GC-waves) across the genome. A minimum of three SNPs were used to define each CNV call. For members within each family processed on the same chip, we were able to further refine boundaries using the PennCNV trio/quartet-based calling algorithm. Samples with a standard deviation of normalized intensity (SDLRR) <0.4 and not more than 300 detected CNVs were included in the analysis. Samples with 0.3<SDLRR<0.4 and a number of detected CNVs between 100 and 300 (n = 34) were visually examined for mosaicism and extremely elevated heterozygosity in BeadStudio V3 software. Due to hemizygosity in males and X-chromosome inactivation in females, the CNV analysis was restricted to autosomes. We used the Kruskal-Wallis rank sum test to compare group specific CNV numbers. Associations in contingency tables were determined using Fisher's exact test.

### Identification of copy number variations concordant with autism spectrum disorders

To identify CNVs that potentially contribute to ASD risk, we performed a multi-step procedure of CNV filtering in the extended families. First, we sought to recognize identical by descent variants by screening for CNVs that segregate with all affected individuals available for testing within the extended family. Next, we excluded CNVs overrepresented in unaffected family members by filtering out CNVs carried by more than one married-in unaffected individual or unaffected child with no clear segregation with affection status, since those CNVs likely segregate in affected individuals by chance alone. To identify CNVs present in the general population that are unlikely to contribute to ASDs, we evaluated each region of interest to determine whether these areas also contained CNVs in our 899 pediatric control samples as well as the Database of Genomic Variants v9 (DGV, http://projects.tcag.ca/variation/). For this assessment, we considered deletions or duplications overlapping by at least 50% of their lengths as the same CNV.

### Validation by real-time PCR copy number assays

Predesigned TaqMan real-time polymerase chain reaction (PCR) copy number assays (Applied Biosystems) were selected within the CNVs chosen for validation using the GeneAssist^TM^ Copy number Assay Workflow Builder (Table S1). Patients carrying CNVs of interest as well as relatives negative for the CNV were evaluated by real-time PCR. Each individual was tested in quadruplicate according to standard protocols. Analysis was performed in the CopyCaller Software v1.0 (Applied Biosystems).

## Results

### Copy number variants identified

In total, 382 samples (97 ASD patients and 285 relatives without a formal ASD diagnosis) were used in the final CNV analysis of the families. Thirteen samples were excluded due to QC failure and an additional fifteen samples were removed because their family branches became uninformative. 838 pediatric control samples survived QC and were also evaluated for CNVs.

Altogether, 23,263 autosomal CNVs at 5,653 distinct genomic locations were identified in the 382 individuals comprising the ASD family cohort. This includes 1,447 homozygous deletions (6.2%), 14,212 single copy deletions (61.0%), 7,534 single copy duplications (32.3%) and 170 homozygous duplications (0.7%). CNVs were detected with an average size of 51.3 kilobases (kb).

We observed a difference in the average number of CNVs between samples originating from different DNA sources (whole blood, saliva, or lymphoblast cell line) as well as variation in samples genotyped on each version of the Illumina BeadChips (Table S2). The genotyping chip design contributes to this variation because the number of sites used for CNV calling is substantially greater on the Human 1 M-DuoV3 than on the Human 1Mv1 C Beadchip (1,199,188 vs. 1,072,821). However, since CNVs of interest were verified for transmission in family members using real-time PCR, these observed differences did not result in an increase of false positive findings in our study.

### Copy number variants concordant with autism spectrum disorders

To determine which CNVs may play a role in ASD risk, we screened for CNVs segregating in all affected individuals within each extended family. We hypothesized that these families would carry a heterozygous genetic component that, while having a moderate to strong influence on ASD, would exhibit incomplete penetrance. We identified overlapping CNV segments shared by every affected individual in each pedigree and examined the transmission from the common ancestor to affected individuals, excluding all recurrent CNVs that did not segregate with affection status. This strategy reduced the initial thousands of copy number variants to a manageable few given that first cousin share 12.5% of their genetic information by chance alone and second cousins share 3.125%.

**Figure 1:**
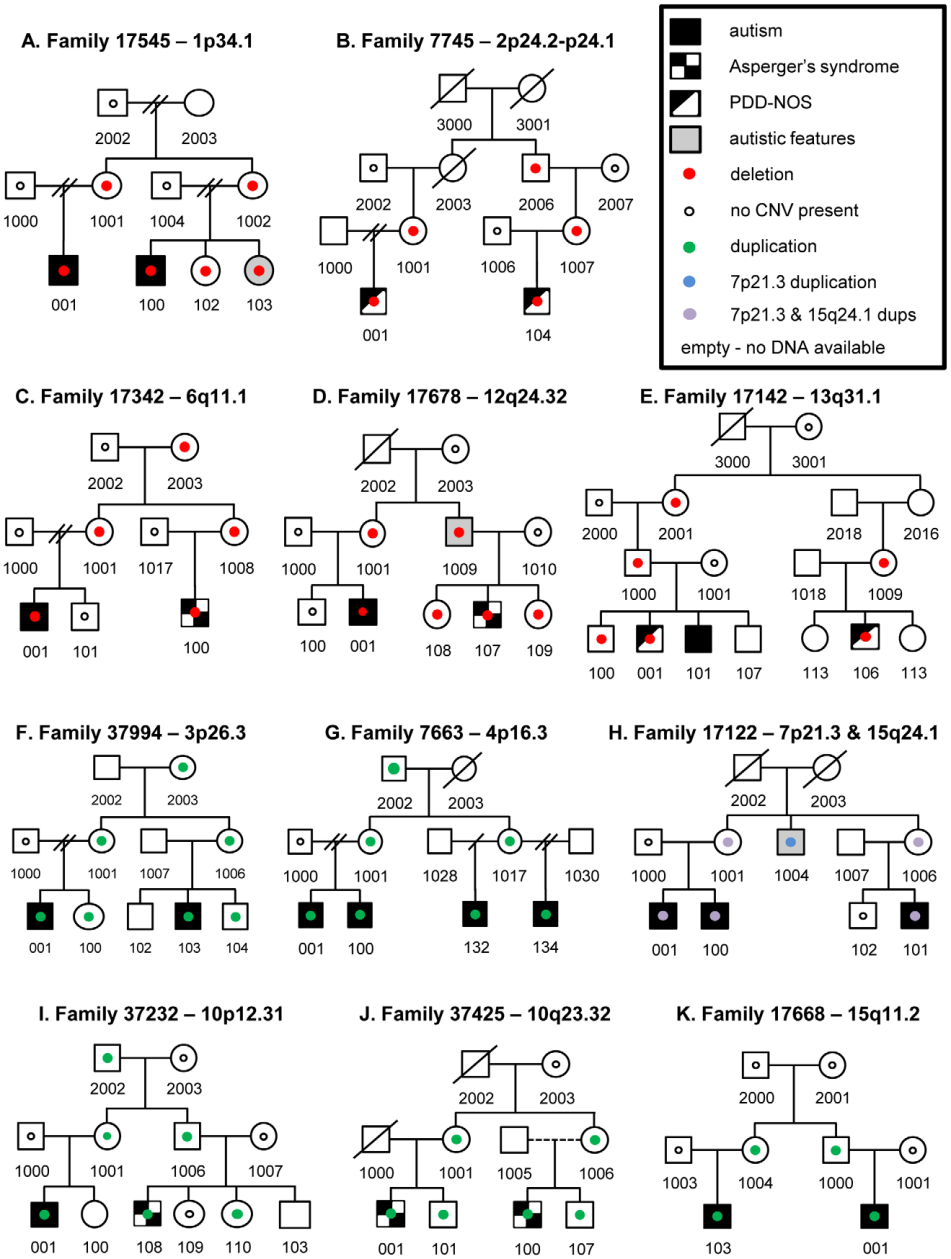
Copy number variations identified in 11 families. A–E) Five extended ASD families had deletions segregating with disease. F–K) Six ASD families carried duplications that segregated with ASD. Family 17122 had two duplications that were inherited in an identical manner (H). Only individuals marked with a small circle had DNA available for testing.

**Figure 2:**
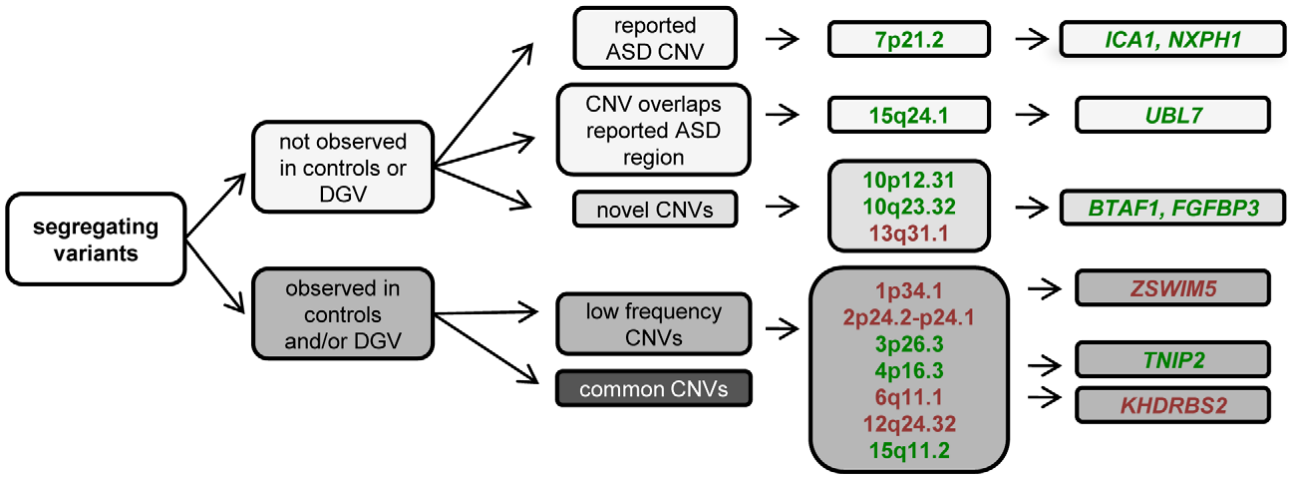
Prioritization of copy number variations of interest. Structural variants were first assessed to determine whether they were present in our control individuals or the Database of Genomic Variants. Literature searches were then performed to determine if the CNVs we identified were previously reported as ASD regions with similar breakpoints or overlay with larger ASD reported regions. Higher priority was given to variation absent in controls and those that fell within regions previously connected to ASDs. Low frequency CNVs were identified in our control HIHG and NBC dataset, but at a frequency below 1.5%. Duplications are in green and deletions are in red.

Twelve CNVs of interest within eleven families of European ancestry were identified and successfully validated using TaqMan real-time PCR copy number assays (Table 1, Figure S1). Our findings include five deletions on 1p34.1, 2p24.2-p24.1, 6q11.1, 12q24.32, and 13q31.1 (Figure 1A–E), along with seven duplications on 3p26.3, 4p16.3, 7p21.3, 10p12.31, 10q23.32, 15q11.2 and 15q24.1 (Figure 1F–K). Five of these CNVs at 7p21.3, 10p12.31, 10q23.32, 13q31.1 and 15q24.1 were not identified in our control samples or in the publicly available DGV database (Figure 2). The microduplications on 7p21.3 and 15q24.1 both segregate in the same four individuals in family 17122 (Figure 1H). While these two duplications were absent from our control dataset and the DGV, they each overlap with ASD regions reported in the literature [35]. Further investigation into this family permitted us to refine the breakpoints for both duplications and sequence the 7p21.3 breakpoints, thereby determining that this CNV was duplicated in tandem [36].

**Table 1:** Validated Copy Number Variations, Nearest Genes and Control Frequencies.

CNV	Cytoband	Boundaries (Build NCBI136/hg18)∧	Estimated length (bp)	Family	Gene Nearest to CNV	Frequency in Caucasian controls (n = 727) [HIHG/NBC]*	Frequency in African American controls (n = 111) [HIHG/NBC]*	Count in DGV (n>9200)
del	1p34.1	chr1:45408389–45411073	2,684	17545	***ZSWIM5***	0.012 [8/1]	0.027 [3/0]	4
	2p24.2–p24.1	chr2:19065745–19100096	34,351	7745	*OSR1*	0	0	5
	6q11.1	chr6:62507037–62519883	12,846	17342	***KHDRBS2***	0	0	2
	12q24.32	chr12:125875006–125881162	6,156	17678	*LOC100128554*	0.007 [1/4]	0	18
	13q31.1	chr13:77912491–77917728	5,237	17142	*POU4F1*	0	0	0
dup	3p26.3	chr3:57010–103697	46,687	37994	*CHL1*	0.003 [2/0]	0	5
	4p16.3	chr4:2704290–2747426	43,136	7663	***TNIP2*** †	0	0	0
	7p21.3	chr7:8144894–8497305∧∧	352,411	17122	***ICA1, NXPH1***	0	0	0
	10p12.31	chr10:21727129–21818381	91,252	37232	*C10orf114*	0	0	0
	10q23.32	chr10:93621936–93730409	108,473	37425	*BTAF1, * ***FGFBP3*** †	0	0	0
	15q11.2	chr15:18411624–18472812	61,188	17668	*GOLGA6L6*	0.004 [3/0]	0.009 [0/1]	>300
	15q24.1	chr15:72538187–72549215∧∧∧	11,028	17122	***UBL7***	0	0	0

∧CNV boundaries correspond to the minimal size detected by Illumina's 1M genotyping array.

∧∧CNV boundaries sequenced in Cukier, et al, 2011.

∧∧∧CNV boundaries correspond to the minimal size refined in Cukier, et al, 2011.

*CNV type reported in controls corresponds to the CNV type (del or dup) identified in ASD extended families.

**Genes in bold** fall within the CNV.

† Whole gene duplication.

HIHG - University of Miami Hussman Institute for Human Genomics control cohort.

NBC - Nashville Birth Cohort.

DGV - Database of Genomic Variants.

**Figure 3:**
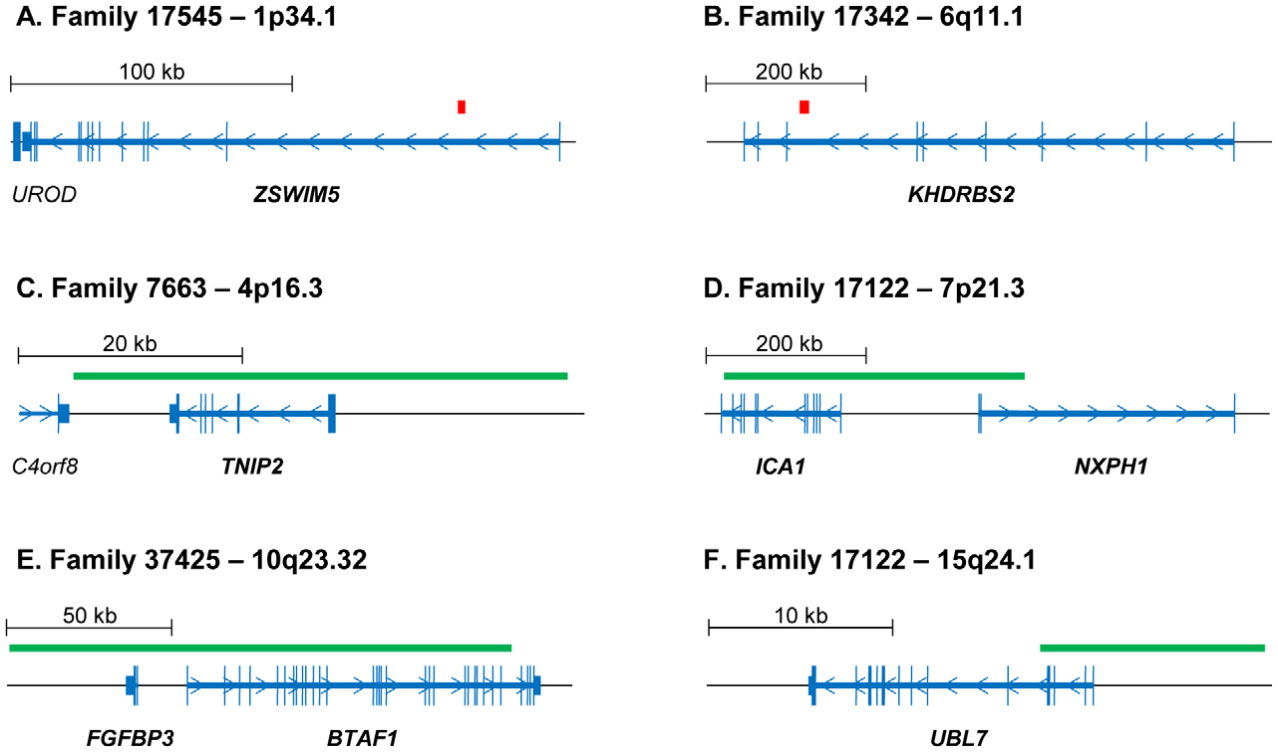
Copy number variations that intersect genes. A,B) Deletions on 1p34.1 and 6q11.1 fall within the intronic regions of *ZSWIM5* and *KHDRBS2*, respectively. C–F) Four duplications on 4p16.3, 7p21.3, 10q 23.2, and 15q24.1 completely encompass the *FGFPB3* and *TNIP2* genes and traverse portions of *BTAF1, ICA1, NXPH1,* and *UBL7*. Deletions are denoted with red bars, duplications marked by green bars and genes are annotated with blue lines. Genes that intersect CNVs are in bold.

Six of the twelve CNVs fall across the following known genes: *BTAF1, FGFPB3, ICA1, KHDRBS2, NXPH1*, *TNIP2, UBL7,* and *ZSWIM5* (Table 1, Figure 2, Figure 3). The deletions on 1p34.1 and 6q11.1 are relatively small (2.7 and 12.8 kb) and located within the intronic regions of *ZSWIM5* and *KHDRBS2*, respectively (Figure 3A,B). In contrast, the four duplications (4p16.3, 7p21.3, 10q 23.2, and 15q24.1) are, for the most part, larger (11.0–352.4 kb) and either span whole genes, *FGFPB3* and *TNIP2*, or include exonic portions of *BTAF1, ICA1, NXPH1,* and *UBL7* (Figure 3C–F).

### Clinical information and CNVs transmission in extended families

To investigate the potential inheritance models of putatively pathogenic CNVs, we evaluated all available family history data (Table S3). Each of the segregating CNVs were found in families with only males affected, although a sister in family 17545 carries the 1p34.1 deletion and was described as having developmental delay and ASD symptoms (Figure 1A, individual 103, Table S3). Five of the CNVs were transmitted maternally while the remaining six were inherited both maternally and paternally. In every family, the CNV passes through individuals without a formal ASD diagnosis, supporting a dominant model of inheritance with incomplete penetrance. Furthermore, the fact that some CNVs are only transmitted maternally suggests possible parent of origin effects.

For all family members negative for a diagnosis of ASD and carrying a CNV of interest, half (19 of 38, 50.0%) have a history of psychiatric or developmental conditions, including ASD symptoms without a formal diagnosis, alcoholism, anxiety disorder, attention deficit and hyperactivity disorder (ADHD), bipolar disorder, major or postpartum depression, obsessive compulsive disorder, oppositional defiant disorder (ODD), and speech disorders. The complete clinical information for these individuals is shown in Table S3. To determine whether CNV carriers had an increased frequency of psychiatric or developmental disorders, we compared the clinical features of twenty CNV carriers to their spouses who did not carry the CNV (individuals specified on Table S3). Twelve of the CNV carriers were found to have one of the clinical diagnoses listed above, while only six of their spouses were diagnosed with one of the above features. When measured by Fisher's exact test, this difference trended towards but did not reach significance (*p* = 0.111). While we believe that the CNVs of interest may confer risk for ASD, our data also suggest that these CNVs may play a more general role in a range of other neuropsychiatric conditions.

## Discussion

Using a unique resource of ASD extended families, twelve loci were found that co-segregate with disease and may be involved in ASD susceptibility. In evaluating the potential pathogenicity of each region, we first prioritized rare structural variants not identified in controls, then proven ASD regions and genomic areas linked to neuropsychiatric disease, and, lastly, on CNVs containing genes with a known, neuronal function (Figure 2). Of the twelve structural variants identified, seven were observed at a low frequency in our control and the DGV datasets and two additional CNVs overlap with those identified in the literature, leaving three novel CNVs unique to our ASD families (Figure 2). Two CNVs fall within regions previously linked to ASD, while five more are appealing candidates due to their locations mapping across or near regions either correlated with neuropsychiatric disease or containing genes related to neuronal processes. Three of the duplications (3p26.3, 7p21.3 and 15q24) also provide further support for the hypothesis that the ubiquitin and cell adhesion pathways are involved in ASD risk [17].

Previous evidence from ASD families intersects with two of the structural variants that we identified: 7p21.3 and 15q24.1. On 7p21.3, duplications of approximately the same size and location have been reported in two additional ASD families [35]. The duplications in all three ASD families fall across *ICA1* (*islet cell autoantigen 1*) and *NXPH1* (*neurexophilin 1*), both appealing candidate genes. Interestingly, *ICA1* localizes to dendrites and plays a role in glutamatergic signaling [37]. Furthermore, the NXPH1 interacts with neurexin Iα, a protein that promotes adhesion between dendrites and axons and has been identified to carry variants in autism patients [38], [39]. The duplication on chromosome 15q24.1 falls within the 1.33 Mb critical region of a previously reported microdeletion and microduplication syndrome found in patients with autistic features, delayed development and mental retardation [36], [40], [41]. At approximately 10 kb, this CNV is much smaller than those previously reported in the region and only falls across a single gene, *UBL7* (*ubiquitin-like 7*) [36]. While *UBL7* is expressed in the brain, little is currently known about its specific function [42].

Three additional loci we identified occur near areas connected to other neuropsychiatric diseases: 3p26.3, 4p16.3 and 12q24.32. Regions on 3p26.3 and 12q24.32 have each been implicated in schizophrenia, with 3p26.3 also being connected to intellectual disability [43]–[45]. While the 3p26.3 microduplication does not intersect any genes, it falls 109 kb upstream of *CHL1* (*cell adhesion molecule with homology to L1CAM*), an intriguing ASD candidate gene. *CHL1* has increased expression in presynaptic terminals and acts to regulate SNARE complexes [46]. Lastly, the 4p16.3 region has been connected to developmental delay, mental retardation, Huntington's disease and Wolf-Hirchhorn syndrome [47], [48]. The variety of features reported in patients carrying CNVs within each region, combined with the pattern of transmission within our families, may demonstrate a variability in penetrance and clinical presentations, possibly in a gender specific manner. For example, there are four male ASD individuals in family 7663 who carry a 4p16.3 duplication, but all six family members with the duplication (individuals 001, 100, 132, 134, 1001, and 1017) were reported to have either a speech or language problem as a child (Table 1, Figure 1G).

The novel 10q23.32 microduplication encompasses a strong ASD candidate, the *fibroblast growth factor binding protein 3* (*FGFBP3*) gene. *FGFBP3* is expressed in fetal brain and binds to the FGFs, which are required throughout brain development, neuronal differentiation and survival [49], [50]. Notably, a transgenic mouse model of a related FGF gene, *FGFR1*, mimics physiological features of schizophrenia such as increased levels of dopamine and sensory impairment [51], [52].

One last intriguing CNV is the novel deletion on 13q31.1. This deletion does not contain a gene, but is located 153.5 kb upstream of the *POU domain, class 4, transcription factor 1* (*POU4F1*) gene, also known as *BRN3A*, an appealing ASD candidate. *POU4F1* plays a role in neuronal development and differentiation through careful timed repression of non-neuronal genes [53], [54]. The 13q31.1 deletion, while not disrupting *POU4F1* directly, may affect the regulation of developmental timing or tissue specific regulation of this gene.

Our approach of using extended ASD families successfully identified rare variants that may be associated with ASD on 7p21.3 and 15q24.1, as well as structural aberrations near regions related to other neuropsychiatric disorders on 3p26.3, 4p16.3 and 12q24.32. These results demonstrate that rare, heritable CNVs play a significant role in ASD etiology. Our findings support the hypothesis that altering the dosage of a gene or its regulatory elements can lead to a severe clinical consequence and brings us one step closer to deciphering the genetic heterogeneity of autism spectrum disorders.

## Supporting Information

Figure S1 **Validation of copy number variations by real-time PCR.** A-K) Results for one assay at each genomic location that was validated. The X axis marks the individual within each family and the Y-axis shows the number of copies. The normal copy number is always two. For those families carrying deletions (A–E), individuals with a copy number of one were confirmed to carry the deletion. For families with duplications (F–G), a copy number of three identifies those individuals with the increase in copy number. Family 17122 (H) shows one assay for each of the duplications it carries on 7p21.3 and 15q24.
(PDF)

Table S1 **TaqMan Real-Time PCR Copy Number Assays.** At least one copy number variation assay was selected to validate each genomic region.
(XLSX)

Table S2 **Number Variations identified in Autism Spectrum Disorder Family Cohort and Control Individuals.** Summary of the number of variations identified, with case and control samples divided by DNA source (blood, cell lines, or saliva) and the type of Illumina SNP array chip used (either version 1Mv1_C or 1M-DuoV3).
(XLSX)

Table S3 **Clinical Information for Families with Validated Copy Number Variations.** This table provides phenotype information for all family members evaluated, with emphasis on ASD symptoms and developmental and neuropsychiatric diagnoses. Deletions and then duplications are listed, organized by chromosomal location.
(XLSX)
