# Supplementary figures and images for: Copy Number Variants in Extended Autism Spectrum Disorder Families Reveal Candidates Potentially Involved in Autism Risk
Source: PLoS One. 2011 Oct 7;6(10):e26049. doi: 10.1371/journal.pone.0026049 (PMC3189231; doi:10.1371/journal.pone.0026049)

**A. Family 17545**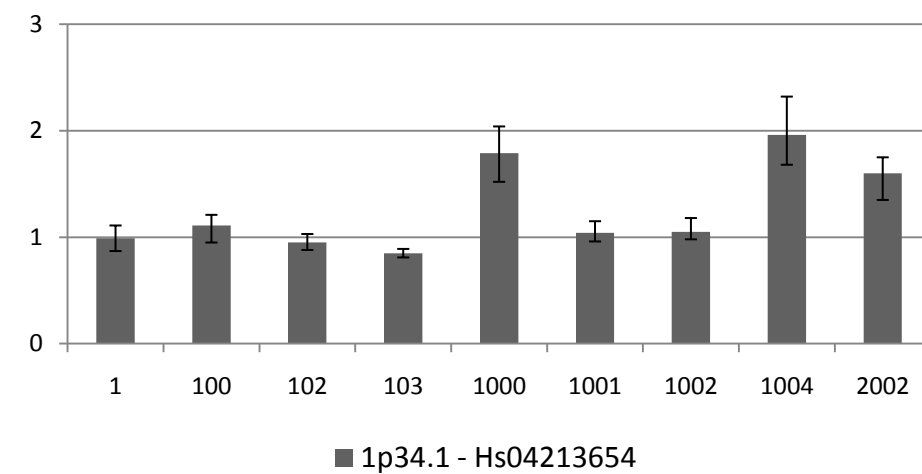**B. Family 7745**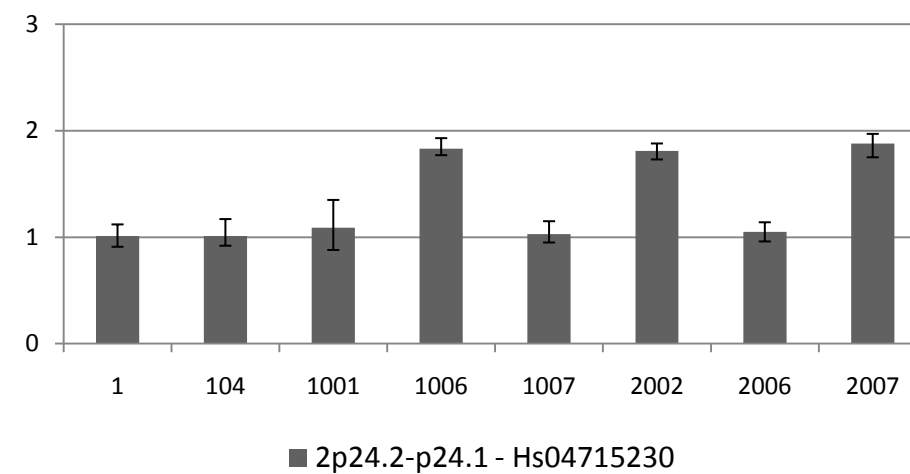**C. Family 17342**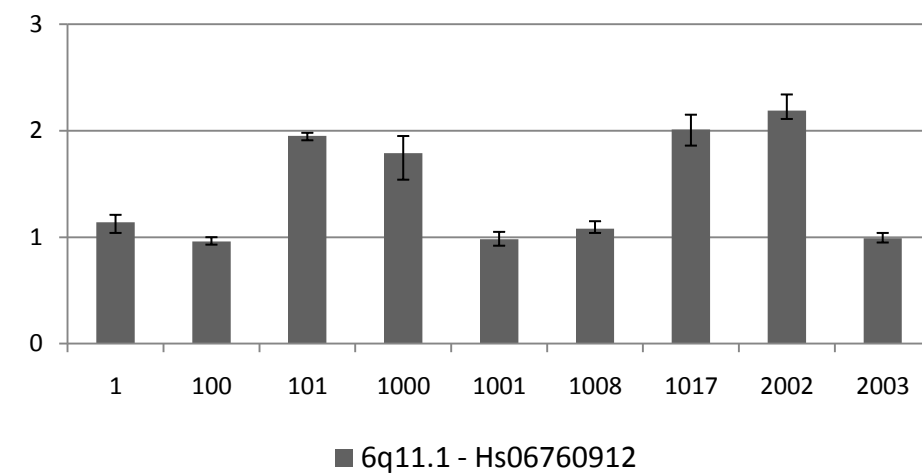**D. Family 17678**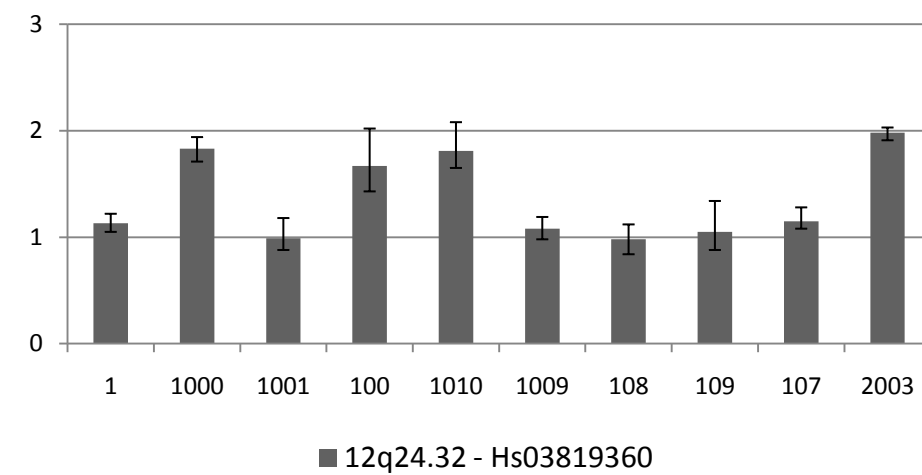**E. Family 17142**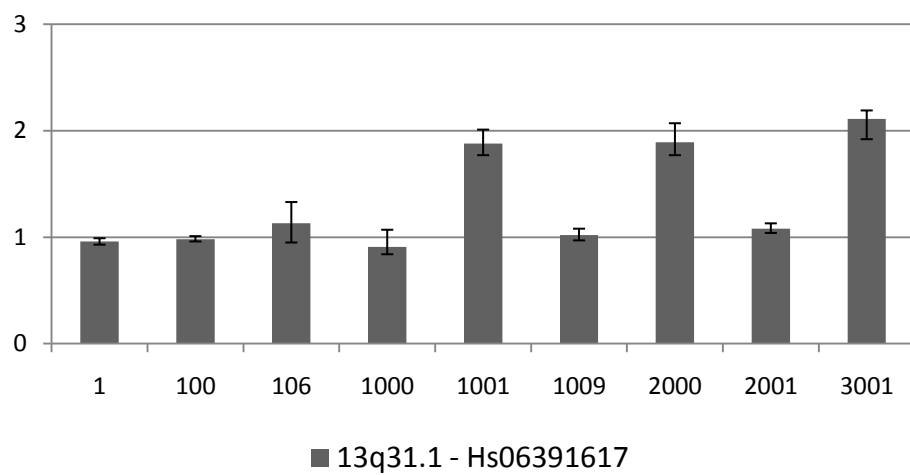**F. Family 37994**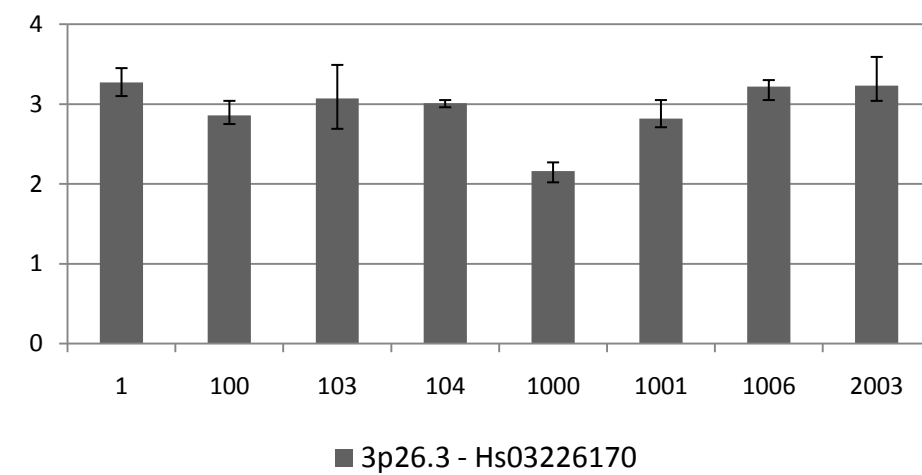**G. Family 7663**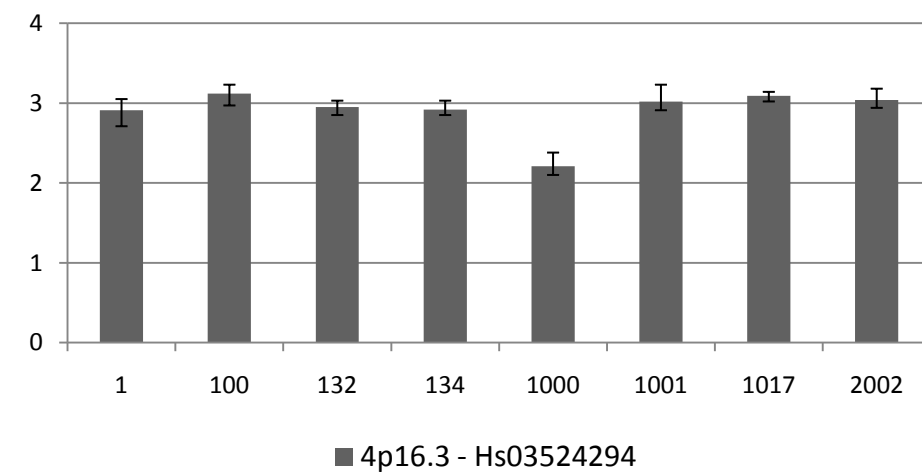**H. Family 17122**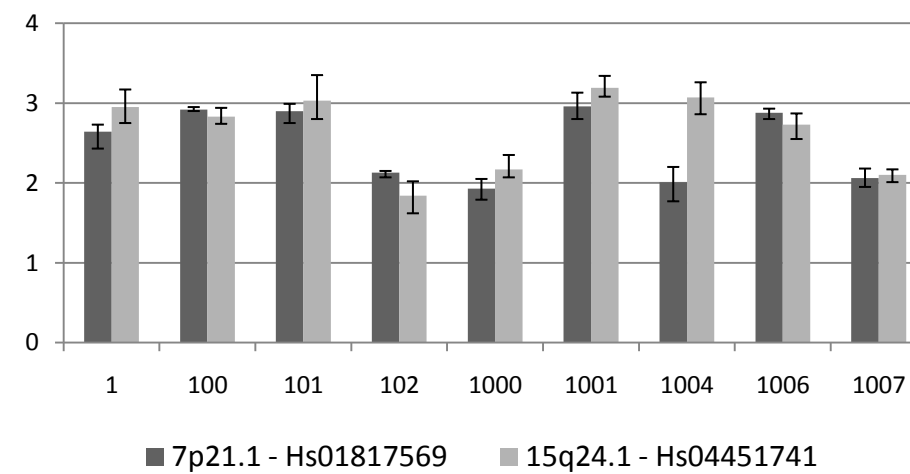**I. Family 37232**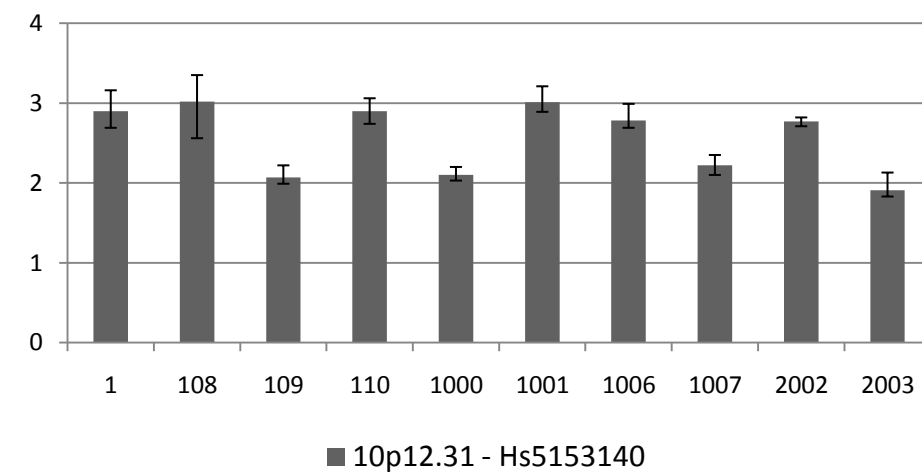**J. Family 37425**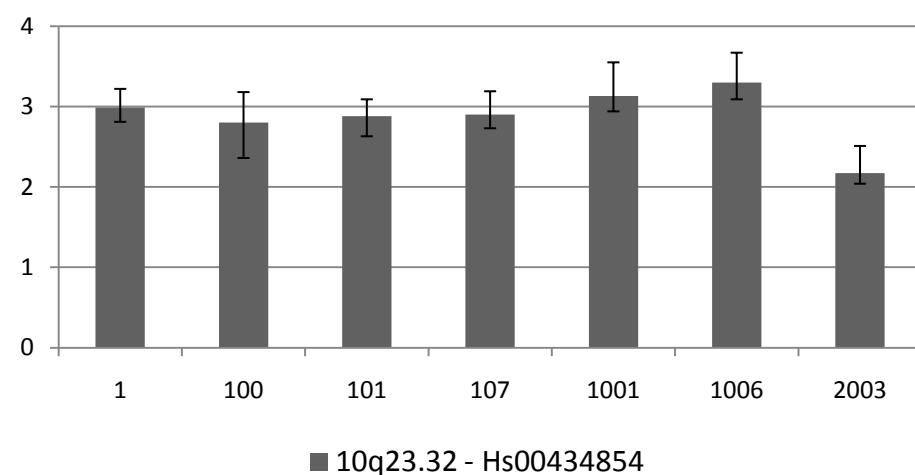**K. Family 17668**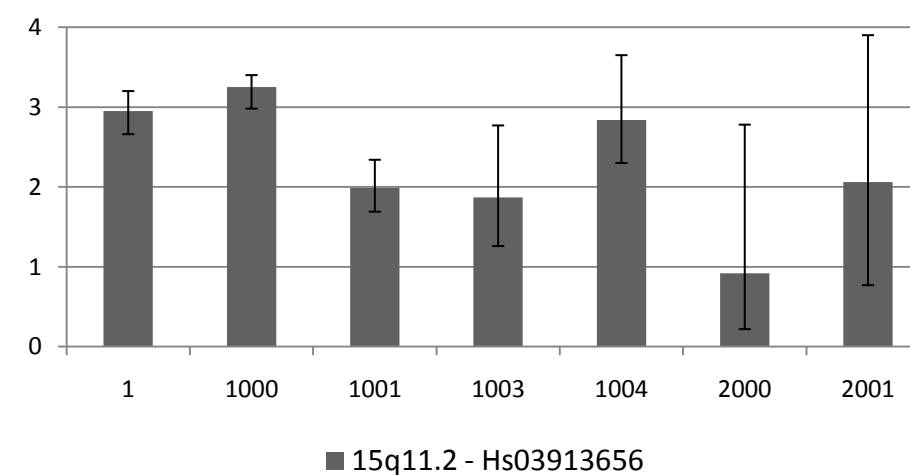

Supplement: Figure S1 — Validation of copy number variations by real-time PCR. A-K) Results for one assay at each genomic location that was validated. The X axis marks the individual within each family and the Y-axis shows the number of copies. The normal copy number is always two. For those families carrying deletions (A–E), individuals with a copy number of one were confirmed to carry the deletion. For families with duplications (F–G), a copy number of three identifies those individuals with the increase in copy number. Family 17122 (H) shows one assay for each of the duplications it carries on 7p21.3 and 15q24. (PDF) [file pone.0026049.s001.pdf]
